# The Diagnostic Performance of Thyroid US in Each Category of the Bethesda System for Reporting Thyroid Cytopathology

**DOI:** 10.1371/journal.pone.0155898

**Published:** 2016-06-27

**Authors:** So Yoon Park, Soo Yeon Hahn, Jung Hee Shin, Eun Young Ko, Young Lyun Oh

**Affiliations:** 1 Department of Radiology and Center for Imaging Science, Samsung Medical Center, Sungkyunkwan University School of Medicine, Seoul, Korea; 2 Department of Pathology, Samsung Medical Center, Sungkyunkwan University School of Medicine, Seoul, Korea; University of Naples Federico II, Naples, Italy, ITALY

## Abstract

We aimed to evaluate the diagnostic performance of thyroid ultrasonography (US) in each category of the Bethesda system and analyze false positive/negative findings using US. This retrospective study included 622 thyroid nodules in 592 patients. The sensitivity, specificity, negative predictive value (NPV), positive predictive value (PPV) and accuracy of US in each category of the Bethesda system were evaluated. False positive/negative cases of US were analyzed. Out of the 622 total thyroid FNAs, 179 (28.8%) were malignant. The malignancy rates for the 6 categories were as follows: I (nondiagnostic): 9.7%, II (benign): 2.5%, III (atypia/follicular lesion of undetermined significance): 37.5%, IV (suspicious for follicular neoplasm): 5.7%, V (suspicious for malignancy): 100%, and VI (malignancy): 100%. The accuracies of US for the 6 categories were 92.5%, 95.6%, 70.8%, 94.3%, 95%, and 92.4% in category order. US showed the lowest sensitivity (50%) in Category IV. Category III demonstrated relatively low sensitivity (66.7%) and specificity (73.3%) due to a high incidence of follicular variant of papillary thyroid carcinoma and a low number of category III nodules. The most optimal performance of US was revealed in Category I with 88.9% sensitivity and 92.9% specificity. In 22 US false positive cases, the most frequent finding was associated with marked hypoechogenicity and the least finding was noncircumscribed margin. The most common US features of 19 false negative cases were circumscribed iso or hypoechoic nodules. These results highlight the excellent diagnostic performance of US in category I of the Bethesda system and the lowest sensitivity of US in category IV. Awareness of US interpreters regarding these pitfalls can minimize false positive/negative diagnoses and prevent unnecessary interventions.

## Introduction

Thyroid ultrasound (US) is the mainstay technique by which thyroid nodules are evaluated. Moreover, a general consensus has now been reached regarding the best US criteria for differentiating benign from malignant nodules [1–3]. The established US features of a malignant nodule include a taller-than-wide shape, an irregular margin, microcalcifications, and marked hypoechogenicity [2,4,5]. Ultrasound-guided fine needle aspiration (FNA) for suspicious thyroid nodules is an accurate and widely used diagnostic method. Cytological results can indicate whether surgery or follow-up is most appropriate for the thyroid nodules. The Bethesda System for Reporting Thyroid Cytopathology (BSRTC) was developed in 2009 to standardize the terminology for interpreting aspiration cytology results [6–8]. The application of BSRTC improved diagnostic accuracy for indeterminate thyroid nodules, leading to higher rates of malignancy detection despite lower rates of thyroidectomies [9]. To manage thyroid nodules, clinicians typically receive two kinds of categorical reports: morphological category, which is based on US, and cytological category, which is based on FNA. It is not yet known whether ultrasound diagnoses have limitations for any of the BSRTC categories, or whether they can provide helpful information for some cytological results. Thus, the purpose of our study was to evaluate the diagnostic performance of thyroid US in each category of the Bethesda System for Reporting Thyroid Cytopathology and analyze false positive/negative findings using US.

## Materials and Methods

### Patients

This retrospective study was approved by the institutional review board of Samsung Medical Center and the requirement for informed consent was waived. This study included thyroid nodules assessed by US-guided FNA between Aug. and Oct. 2010 at our institution. Our institution served about average 8000 US-guided FNAs per year by radiologists at the study period. Generally, it seems that incidence of the malignancy of thyroid nodules by FNAs performed in our hospital is about 25–30%. This incidence rate is relatively high because our hospital is a tertiary referral center. All nodules were categorized based on the Bethesda system. We retrospectively reviewed the pathology and US reports of each patient. Patient records were analyzed anonymously. A total of 1353 FNAs in 1345 patients were performed during the study period. From these, nonthyroidal lesion (*n* = 93), nodules smaller than 0.5cm (*n* = 155), and nodules with no acceptable follow-up or operation (*n* = 483) were excluded. Finally, 622 nodules were selected from 592 patients who were followed up for at least 2 years or underwent surgery. Statistical analysis was performed on these 622 thyroid nodules.

### Thyroid Ultrasound and Image Analysis

Thyroid US was performed at a frequency range of 7 to 15 MHz on an iU22 (Vision 2010; Philips, Seattle, WA, USA) by one of 7 radiologists. All radiologists had 1 to 11 years of experience in thyroid imaging.

The US features of the thyroid nodules were prospectively analyzed by the radiologist who performed the US examination. All nodules were classified into one of three categories (benign, indeterminate, and malignant) according to the Korea Society of Thyroid Radiology (KSThR) guidelines [2]. The KSThR guidelines take into account the internal components, echogenicity, margin, calcification, shape, and orientation of the thyroid nodule and categorized thyroid nodules in to three US diagnosis (Table 1). A taller-than-wide shape, a spiculated or irregular margin, marked hypoechogenicity, microcalcifications, and macrocalcifications are all findings suggestive of malignancy [2]. The presence of at least one of these findings defined a nodule as a malignant nodule. In contrast, simple cysts, predominantly cystic or cystic nodules with reverberating artifacts, and nodules with a spongiform appearance (especially with intervening isoechoic parenchyma) were defined as benign nodules. Indeterminate nodules had neither malignant nor benign features; iso-, hypo- or hyperechogenecity, ovoid-to-round shape, irregular shape, smooth or ill-defined margin, and rim calcification.

**Table 1:** US features of thyroid nodules based on the Korea Society of Thyroid Radiology (KSThR) guidelines.

	US features
Probable benign	Simple cyst, predominantly cystic, cystic nodule with reverberation artifact, spongiform nodule
Indeterminate	Isoechogenecity, hypoechogenecity (compared to thyroid parenchyma), hyperechogenecity, ovoid-to-round shape, irregular shape, smooth or ill-defined margin, rim calcification
Suspicious malignant*	Taller than wide shape, speculated margin, marked hypoechogenicity (compared to strap muscle), microcalcification (<1mm), macrocalcification (>1mm)

*: The presence of at least one of the findings for malignancy defines a nodule as a suspicious malignant nodule

### Cytological Analysis

US-FNA was performed by one of the seven trained radiologists who conducted the US examinations. US-FNA was performed manually with a 23-gauge needle attached to a 2-mL disposable syringe. On average, 1–2 passes were performed for each nodule. Aspirates were smeared onto a glass slide,immediately fixed in 95% alcohol for Papanicolaou and hematoxylin and eosin staining. No Giemsa stain, liquid-based cytology or cytobloc were performed. One of six cytopathologists interpreted the FNA specimens. All cases were reported using a six-tiered diagnostic system according to the Bethesda System for Reporting Thyroid Cytopathology [6]. Nodules were classified into the following cytological categories: (1) nondiagnostic or unsatisfactory (Bethesda System I), (2) benign (Bethesda System II), (3) atypia of undetermined significance (AUS)/follicular lesion of undetermined significance (FLUS) (Bethesda System III), (4) follicular neoplasm or suspicious for a follicular neoplasm (Bethesda System IV), (5) suspicious for malignancy (Bethesda System V), and (6) malignant (Bethesda System VI).

### Data and Statistical Analysis

All thyroid nodules were categorized according to their US features and also according to their cytopathologic results. Although nodules were classified into three groups according to their US results, nodules identified as indeterminate by US were treated as benign for all subsequent statistical analysis. Thus, statistical analysis was performed on two US categories, probably benign and malignant.

Lesions were considered to be cytopathologically benign if they met at least one of the following conditions: 1] pathologically confirmed as benign by thyroidectomy or core needle biopsy; 2] US follow-up for at least 2 years with either no interval change or a decrease in size after an initial benign cytology finding; and 3] benign cytology by more than two FNAs. Nodules were defined as malignant if they were confirmed as malignant thyroid carcinoma by two serial FNAs or by thyroidectomy.

Data were analyzed using the Statistical Package for the Social Sciences for Windows (Version 17.0.1, SPSS, Chicago, IL, USA). In each category of the Bethesda system, the sensitivity, specificity, positive predictive value (PPV), negative predictive value (NPV), and accuracy of ultrasonography were calculated using the McNemar test. Fisher's Exact Test for Count Data was used to determine whether the differences in accuracy between the 6 Bethesda groups were significant. A *P* value < 0.05 was considered to be statistically significant.

## Results

A total of 592 patients with 622 thyroid nodules (male: 119, female: 473) were included in this study. The mean age was 49.8 years old (range, 14–86 years) and the mean nodule size was 1.61 cm (range, 0.6–7.0cm).

Among all 1105 thyroid nodules which underwent FNA with or without follow up or surgery, the distribution of the Bethesda category was as follows: 163 (14.8%) in category I, 651 (58.9%) in category II, 37 (3.3%) in category III, 45 (4.1%) in category IV, 30 (2.7%) in category V and 179 (16.2%) in category VI. Among them, 622 thyroid nodules which were finally included in statistical analysis were classified into the Bethesda categories as follows: 93 (15.0%) in category I, 319 (51.3%) in category II, 24 (3.9%) in category III, 35 (5.6%) in category IV, 20 (3.2%) in category V, and 131 (21.0%) in category VI.

**Fig 1:**
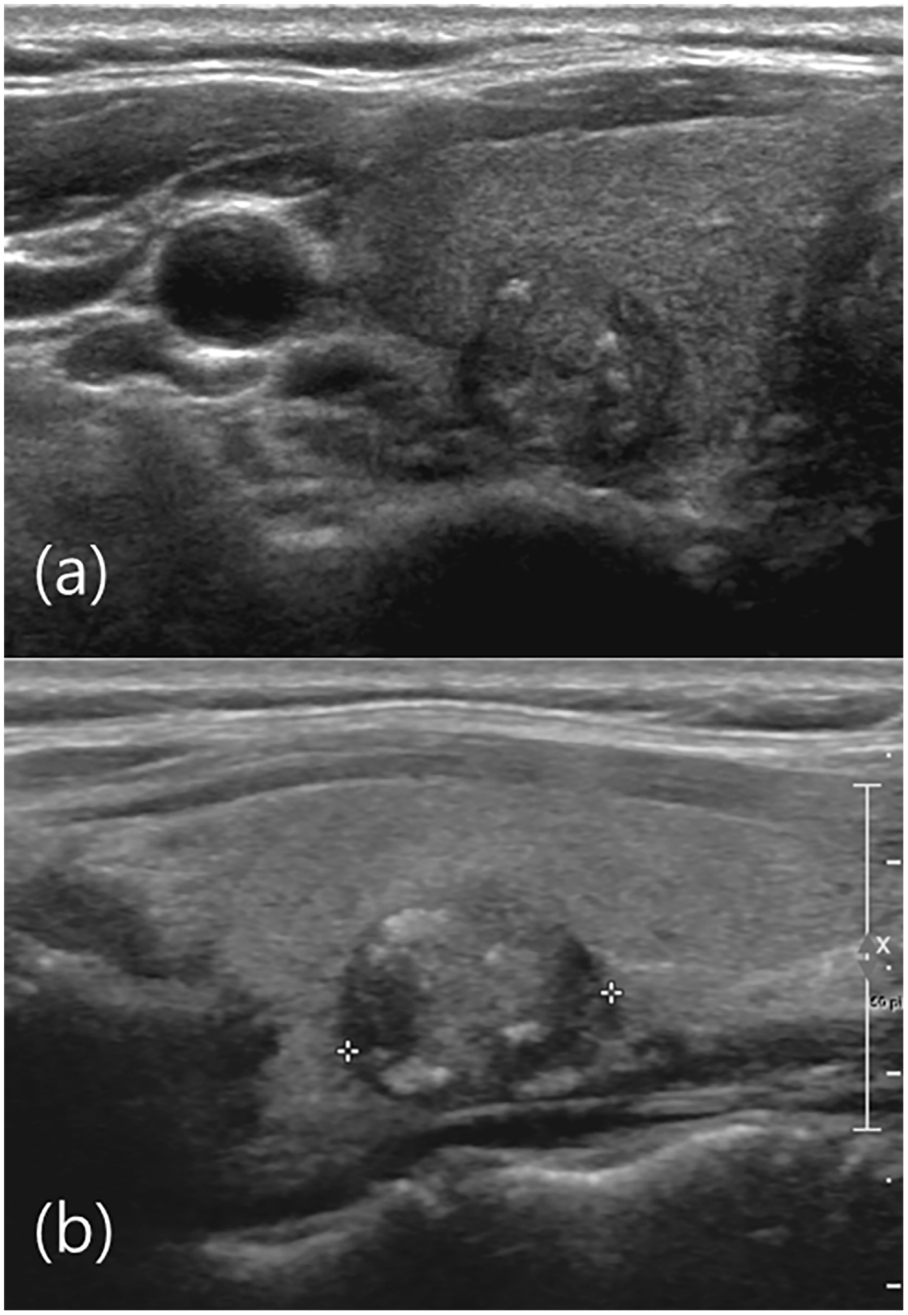
US image of right thyroid gland of a 46-year-old male. (a) Axial view show microlobulated isoechoic nodule with microcalcifications. (b) Sagittal view show a 1.3 cm microlobulated isoechoic nodule with microcalcifications (crosses) in the mid portion of the right thyroid gland. The US diagnosis was malignant and the FNA result was Bethesda category I (non-diagnostic). Total thyroidectomy was performed and the pathological result was papillary thyroid cancer. US was highly sensitive in identifying nodules diagnosed as Bethesda category I.

The proportions of US diagnoses (benign, indeterminate, and malignant) in each Bethesda category are listed in Table 2 (Fig 1). In total, 440 (70.7%) thyroid nodules were classified as probably benign by US (“US probably benign”) and 182 (29.3%) thyroid nodules were malignant by US (“US malignant”). Out of all the US probably benign nodules, 68.9% (303/440) were classified in Bethesda category II. Out of the 622 thyroid nodules, 182 (29.3%) were ultimately classified as US malignant. Out of all the US malignant nodules, 66.5% (121/182) were classified in Bethesda category VI and 76.9% (140/182) were classified in Bethesda category V and VI.

**Table 2:** Distributions of US diagnoses in each bethesda category.

Bethesda Category	No. of nodules	US probably benign		US malignant
	*n* = 622	*n* = 440 (70.7%)		*n* = 182 (29.3%)
		US benign	US indeterminate	
		*n* = 285 (45.8%)	*n* = 155 (24.9%)	
**I**	93 (15.0%)	54 (8.7%)	25 (4.0%)	14 (2.3%)
**II**	319 (51.3%)	214 (34.4%)	89 (14.3%)	16 (2.6%)
**III**	24 (3.9%)	6 (1.0%)	8 (1.3%)	10 (1.6%)
**IV**	35 (5.6%)	8 (1.3%)	25 (4.0%)	2 (0.3%)
**V**	20 (3.2%)	0	1 (0.1%)	19 (3.1%)
**VI**	131 (21.0%)	3 (0.5%)	7 (1.1%)	121 (19.5%)

Classification of a nodule as benign was determined by operation or core needle biopsy in 55 nodules, two FNAs in 112 nodules, and US follow-up after FNA with benign result in 276 nodules. Of the malignant nodules, 172 were confirmed by surgery and 7 were confirmed by two serial FNAs or core needle biopsy. These 3 patients did not undergo surgery due to refusal of the operation and having aggressive malignancy of another organ. Out of the 179 malignant nodules, 171 were papillary thyroid carcinomas (PTC), 4 were follicular thyroid carcinoma (FTC). 2 were medullary thyroid carcinoma (MTC) and 2 were diffuse large B-cell lymphoma.

**Fig 2:**
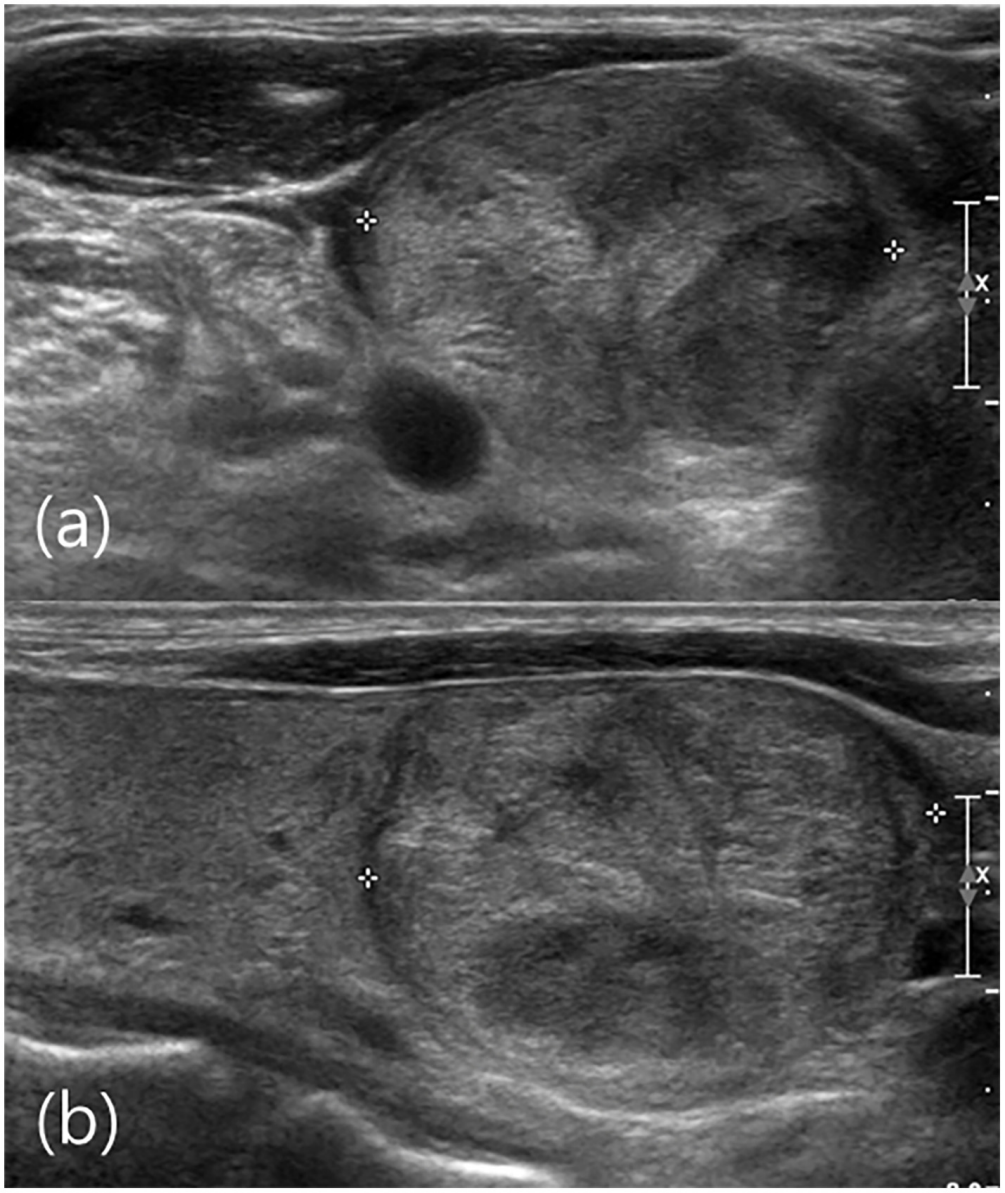
US image of right thyroid gland of a 51-year-old male. (a) Axial view show a circumscribed isoechoic nodule (crosses). (b) Sagittal view a 2.9 cm circumscribed isoechoic nodule (crosses) is visible in the lower portion of the right thyroid gland. The US diagnosis was probably benign and the FNA result was Bethesda category IV (follicular neoplasm). Follicular thyroid carcinoma was revealed by total thyroidectomy. US was not informative for this Bethesda category IV nodule.

The final cytopathological results in each Bethesda category are shown in Table 3. The malignant rates for the 6 categories were as follows: Bethesda System I: 9.7% (9/93), Bethesda System II: 2.5% (8/319), Bethesda System III: 37.5% (9/24), Bethesda System IV: 5.7% (2/35), Bethesda System V: 100% (20/20), and Bethesda System VI: 100% (131/131) (Fig 2). All Bethesda category V and VI nodules were ultimately classified as malignant, with no false positives detected.

**Table 3:** Final cytopathological results in each Bethesda category.

Bethesda category	No. of nodules	Final benign	Final malignant
	*n* = 622	*n* = 443 (71.2%)	*n* = 179 (28.8%)
**I**	93 (11.5%)	84 (90.3%)	9 (9.7%)
**II**	319 (52.8%)	311 (97.5%)	8 (2.5%)
**III**	24 (4.81%)	15 (62.5%)	9 (37.5%)
**IV**	35 (6.25%)	33 (94.3%)	2 (5.7%)
**V**	20 (5.77%)	0 (0%)	20 (100%)
**VI**	131 (18.7%)	0 (0%)	131 (100%)

We calculated the sensitivity, specificity, positive predictive value (PPV), negative predictive value (NPV), and accuracy of the US diagnosis in each Bethesda category (Table 4). The sensitivities of US in categories I, V and VI were 88.9%, 95% and 92.4%, respectively, while the sensitivities in categories II, III and IV were 62.5%, 66.7% and 50%, respectively. The US showed the lowest sensitivity in Category IV. The specificity was also high in Bethesda category I (92.9%) and could not calculate the specificity of category V and VI because all Category V and VI thyroid nodules were final malignant. The specificities were also high in category II and IV, 96.5% and 97.0%, respectively but relatively low in Bethesda category III (73.3%). PPVs were 100% in category V and VI and those of other categories were less than 60%. NPVs were high in category I, II and IV, 98.8%, 99.0% and 97.0%, respectively. The accuracies of US for the 6 categories were 92.5%, 95.6%, 70.8%, 94.3%, 95.0%, and 92.4% (in category order). The most optimal performance of US was revealed in Category I with 88.9% sensitivity and 92.9% specificity.

**Table 4:** Performance of ultrasonography in each Bethesda category.

Bethesda category	Sensitivity (%)	Specificity (%)	PPV (%)	NPV (%)	Accuracy (%)
**Category I**	88.9	92.9	57.1	98.8	92.5
**Category II**	62.5	96.5	31.3	99.0	95.6
**Category III**	66.7	73.3	60	78.6	70.8
**Category IV**	50	97.0	50	97.0	94.3
**Category V**	95	-	100	0	95.0
**Category VI**	92.4	-	100	0	92.4

False positive cases which were considered malignant on US were 22. The most frequent finding was associated with marked hypoechogenicity and the least finding was noncircumscribed margin. Thirteen (59%) of 22 had only one suspicious feature. False negative cases were 19. The reasons for FNA in 19 false negative US cases confirmed later were as follows; nodules larger than 1cm with US indeterminate (11 cases), interval increased size (6 cases), and PET uptakes (2 cases). Three FTCs showed probably benign US features and FNA results were Category I, II and IV. The PTC arising from follicular adenoma, 4 follicular variants of PTC showed probably benign US features and FNA results were Category II and III. Eleven PTCs with FNA results V and VI showed probably benign US features. The most common features of false negative showed circumscribed iso or hypoechoic nodules.

## Discussion

Thyroid US and US-guided FNA are the two leading diagnostic tools for evaluating thyroid nodular disease. The decision of whether to conduct surgery or to perform follow-up is taken based on thyroid US results together with cytological findings [10].

Due to the lack of information regarding the extent to which US and cytological reports are correlated, it may be difficult for physicians and surgeons to make treatment decisions. Several studies have evaluated the extent to which US diagnoses correlate with cytological results [11–16]. Lee at al. evaluated the usefulness of a combined categorical reporting system, including both US and cytological results, for deciding when repeat US-guided FNA should be performed [11]. Some investigators reported the incidence of thyroid cancer among cases with non-diagnostic (Bethesda category I) cytology and additionally evaluated the criteria for selecting those for repeat FNA according to US features [12,16]. Kim et al. and Rosario et al. reported the diagnostic efficacy of US in evaluating thyroid nodules, especially for Bethesda category III nodules [13,15]. In our study, we evaluated the diagnostic performance of US in each of the 6 Bethesda categories.

In our study, US showed the most optimal performance in Bethesda category I with a sensitivity of 88.9% and a specificity of 92.9%. Only one Bethesda category I nodule with the confirmation as FTC after operation was counted as false negative in US. This finding indicated that US can play an important role in determining further management for cytologically non-diagnostic thyroid nodules. Specifically, US follow-up rather than re-aspiration is recommended for US benign-looking nodules with Bethesda I results according to high sensitivity, specificity and accuracy of US in Bethesda category I thyroid nodules. This recommendation is consistent with that of Lee et al., who also recommended follow-up for US benign non-diagnostic nodules. This conclusion was based on the high possibility of other non-diagnostic FNA results, which are of little clinical relevance [11]. Moon et al. also recommended follow up rather than re-aspiration if Bethesda category I nodules have one or less suspicious US feature or nodules with cystic portion greater than 50% [16].

Bethesda category III is cytologically indeterminate and its rate of malignancy has been reported to range from 5–22.6% [15,17]. The recommended management for category III is clinical correlation and a repeated FNA at an appropriate interval. However, many reports have cited a risk of malignancy that should be considered in the management of Bethesda III nodules. This risk depends on the particular physician and institution and is influenced by clinical observations, repeat FNAs, core-needle biopsies, and surgeries [15,17]. To overcome the diagnostic limitations of cytology in indeterminate categories, many studies have evaluated the ability of thyroid US to predict malignancy for Bethesda category III nodules, with the aim of identifying management guidelines for these lesions [2,17–20]. In our study, US had a sensitivity of 66.7%, a specificity of 73.3%, a PPV of 60.0%, and an NPV of 78.6% in Bethesda category III nodules. Rosario et al. recently reported prospective study for clinical, laboratory, ultrasonographic, and cytological predictors of malignancy in Bethesda category III thyroid nodules. US sensitivity, specificity, PPV and NPV were 79.4%, 90.5%, 71% and 93.5%, respectively [15]. Several other studies also suggested the usefulness of US for evaluating malignancy of Category III nodules [11,13,17]. Sensitivity and specificity were relatively low in our study compared to previous studies, probably because of low number of category III nodules (4.8%). Three category III nodules showed false negative result of US which led to relatively low sensitivity in our study, and all those 3 nodules were follicular variant of PTC. Follicular variants of PTC are reported to have relatively benign appearance on sonography that is more similar to those of follicular neoplasm than PTCs and this might be the reason of false negative US findings [21].

The accuracies of US in Bethesda categories III is relatively low (70.8%) compared to that of Category I (92.5%), II (95.6%), IV (94.3%), V (95.0%) and VI (92.4%). We speculate that the low accuracy of US in Bethesda category III nodules resulted from our classification of US indeterminate nodules treated as US probably benign nodules. If US indeterminate nodules would have been considered to be US malignant nodules, better sensitivity and accuracy would have been achieved. However, in contrast to nodules with malignant features, US indeterminate nodules should not be managed under strict guidelines because most thyroid carcinomas are not aggressive and have a good prognosis.

Meanwhile, the sensitivity and PPV in categories IV were 50% and 50%, respectively. Although the specificities and NPVs of US for nodules with cytologically suspicious for follicular neoplasm (IV) were high. This result indicates that the current US morphologic guidelines for follicular neoplasm are of limited value. The current US features mainly reflect papillary thyroid cancer, which limits the sensitivity of US in Bethesda category IV nodules. Further studies with pathology-radiology correlation are needed for follicular neoplasm of thyroid gland.

Among 22 false positive cases at US, the most frequent finding was marked hypoechogenicity and the least finding was noncircumscribed margin. Major reason for false positive cases was because nodules were interpreted as marked hypoechoic due to uncontrolled sonic gain. At follow-up of these nodules, they were not marked hypoechoic under control of proper gain. Careful adjustment of sonic gain is crucial for appropriate diagnosis of thyroid nodule and increase the efficacy of US. Benign thyroid nodules showed irregular or noncircumscribed margin when previously existing fluid component had disappeared causing shrinkage of thyroid nodules.

Comparing the 2011 KSThR guidelines to 2015 American thyroid association (ATA) guidelines, KSThR US “probable benign” correlates to US “very low suspicion” and “benign” of ATA guidelines. KSThR US “indeterminate” correlates to US “intermediate suspicion” and “low suspicion” of ATA. KSThR US “suspicious malignant” correlates to “high suspicion” of ATA. The major difference between two guidelines divided indeterminate and probable benign categories into more detailed categorizations. ATA recommended FNA at different size in “intermediate suspicion” and “low suspicion” (> 1cm and > 1.5cm, respectively), whereas KSThR recommended FNA in US “suspicious malignant”nodules > 0.5cm and US indeterminate nodules ≥ 1cm. Another difference is emphasizing rim calcified lesion. KSThR guidelines categorized rim calcified nodules into indeterminate category and commented that the presence of a hypoechoic halo and rim disruption are more suggestive of malignancy. In ATA guidelines, rim calcified nodules with small extrusive soft tissue component were categorized into “high suspicious” category. The other difference is macrocalcifications. KSThR guidelines categorized macrocalcifications as US “suspicious malignant”. In ATA guidelines, macrocalcifications have same malignancy risk as microcalcifications if they are combined with microcalcifications [2,22].

Our present study did not investigate the genetic abnormalities of thyroid nodules. Many investigators have reported that detection of RET/PTC, TRK and BRAF(V600E) in FNAB specimens is proposed as a diagnostic adjunctive tool in the evaluation of thyroid nodules with suspicious cytological findings. [23–25]. The combination of US findings, biopsy and genetic study is the most reliable triage as the current options for the evaluation of undetermined thyroid nodules.

Our study did have several limitations. First, this was a retrospective study based on radiologic and pathologic reports. Second, the US and FNA analyses of the 622 nodules were not performed by a single radiologist. Although all the radiologists who performed the US and FNA analyses were extensively trained, inter-assessor variation could have led to different US findings for nodules that are not easily classified. We did not calculate the interobserver variability between individuals. However, we have a system that can minimize the difference. Unexperienced (less than 2 years) radiologists have received the confirmation about all cases with equivocal or indeterminate US features from experienced radiologists in the next room or in real time. Each radiologist has a chance to control the threshold through intradepartment conference. Moreover, differences in FNA skill levels could have increased the number of non-diagnostic results. However, this scenario is representative of clinical practice.

In conclusion, these results highlight the excellent diagnostic performance of US in category I of the Bethesda system and the lowest sensitivity of US in category IV. Awareness of US interpreters regarding these pitfalls can minimize false positive/negative diagnoses and prevent unnecessary interventions.
